# The Fast Track FIT study: diagnostic accuracy of faecal immunochemical test for haemoglobin in patients with suspected colorectal cancer

**DOI:** 10.3399/BJGP.2020.1098

**Published:** 2021-07-13

**Authors:** James L Turvill, Daniel Turnock, Dan Cottingham, Monica Haritakis, Laura Jeffery, Annabelle Girdwood, Tom Hearfield, Alex Mitchell, Ada Keding

**Affiliations:** Department of Gastroenterology, York and Scarborough Teaching Hospitals NHS Foundation Trust, York.; Department of Gastroenterology, York and Scarborough Teaching Hospitals NHS Foundation Trust, York.; Macmillan GP Cancer and End of Life lead, Vale of York Clinical Commissioning Group, West Offices Station Rise, York.; Department of Research and Development, York and Scarborough Teaching Hospitals NHS Foundation Trust, York.; Department of Research and Development, York and Scarborough Teaching Hospitals NHS Foundation Trust, York.; Department of Research and Development, York and Scarborough Teaching Hospitals NHS Foundation Trust, York.; Department of Research and Development, York and Scarborough Teaching Hospitals NHS Foundation Trust, York.; Department of Health Sciences, Faculty of Sciences, University of York, York.; Department of Health Sciences, Faculty of Sciences, University of York, York.

**Keywords:** colorectal neoplasms, early detection of cancer, feces, predictive value of tests

## Abstract

**Background:**

The faecal immunochemical test (FIT) is now available to support clinicians in the assessment of patients at low risk of colorectal cancer (CRC) and within the bowel cancer screening programme.

**Aim:**

To determine the diagnostic accuracy of FIT for CRC and clinically significant disease in patients referred as they were judged by their GP to fulfil National Institute for Health and Care Excellence guideline 12 (NG12) criteria for suspected CRC.

**Design and setting:**

Patients referred from primary care with suspected CRC, meeting NG12 criteria, to 12 secondary care providers in Yorkshire and Humber were asked to complete a FIT before investigation.

**Method:**

The diagnostic accuracy of FIT based on final diagnosis was evaluated using receiver operating characteristics analysis. This permitted a statistically optimal cut-off value for FIT to be determined based on the maximisation of sensitivity and specificity. Clinicians and patients were blinded to the FIT results.

**Results:**

In total, 5040 patients were fully evaluated and CRC was detected in 151 (3.0%). An optimal cut-off value of 19 µg Hb/g faeces for CRC was determined, giving a sensitivity of 85.4% (95% confidence interval [CI] = 78.8% to 90.6%) and specificity of 85.2% (95% CI = 84.1% to 86.2%). The negative predictive value at this cut-off value was 99.5% (95% CI = 99.2% to 99.7%) and the positive predictive value 15.1% (95% CI = 12.8% to 17.7%). Sensitivity and specificity of FIT for CRC and significant premalignant polyps at this cut-off value were 62.9% (95% CI = 57.5% to 68.0%) and 86.4% (95% CI = 85.4% to 87.4%), respectively; and when including all organic enteric disease were 35.7% (95% CI = 32.9% to 38.5%) and 88.6% (95% CI = 87.5% to 89.6%), respectively.

**Conclusion:**

FIT used in patients fulfilling NG12 criteria should allow for a more personalised CRC risk assessment. FIT should permit effective, patient-centred decision-making to inform the need for, type, and timing of further investigation.

## INTRODUCTION

The National Institute for Health and Care Excellence (NICE) has issued guidance to help GPs identify those patients at increased risk of colorectal cancer (CRC) (NICE guideline 12 [NG12]).^1^ These recommendations are largely symptom-based, modified by age. The guidance is underpinned by a ‘2-week wait’ referral pathway to secondary care and other national targets for timeliness in treatment.^2^ Delivering these diagnostic and treatment targets has proven very challenging for secondary care providers.^3^^–^^6^ There has been a yearly increase in ‘2-week wait’ referrals of patients with suspected CRC, but despite this, the number of CRC cases detected through this pathway has changed little.^7^ Since the prevalence of CRC in this cohort of patients is 3%–5%, large numbers of often older and frail patients undergo unnecessary, invasive, unpleasant, and expensive investigations, which are not without risk of complication.^8^ Because investigative capacity (notably colonoscopy and computed tomography [CT] scans) is constrained, the investigative burden placed on secondary care by NG12 has had the indirect effect of limiting the availability of investigative resource to support other CRC diagnostic pathways.^9^^–^^11^

Faecal immunochemical test (FIT), a quantitative test for human haemoglobin in faeces, has been recommended to guide referral of patients who are considered to be at ‘low risk’ of CRC.^12^^–^^14^ A FIT ≥10 µg Hb/g faeces escalates the patient into the ‘2-week wait’ pathway. FIT is also used in the bowel cancer screening programme (BCSP) where the cut-off value is set higher, at 120 µg Hb/g faeces.^15^ There is increasing interest in whether FIT has a role in decision making for all patients fulfilling NG12 criteria. It has been postulated in previous studies that FIT might refine and improve the diagnostic pathway for CRC in these patients. Initial studies suggested that FIT has a sensitivity and specificity of 84.6% and 88.5%, respectively, for CRC in the context of patients fulfilling the NG12 referral guidance.^16^

A diagnostic accuracy study of FIT in patients referred through the ‘2-week wait’ pathway with suspected CRC was undertaken. FIT was provided before secondary care investigation and assessed against final diagnosis for CRC (primary outcome) and for significant premalignant polyps, inflammatory bowel disease (IBD), and other organic enteric disease (OED) combined (secondary outcomes). Additionally, the study sought to identify all clinically significant disease detected in those referred patients in order to obtain a true picture of the benefits of the current NG12 guidance.

**Table:** How this fits in

The role of the faecal immunochemical test (FIT) in the assessment of patients at high risk of colorectal cancer (CRC) is uncertain. FIT has a high sensitivity and specificity for CRC with an area under the curve of 0.89. Some patients in whom FIT sufficiently alters their risk should no longer be investigated within the ‘2-week wait’ pathway. However, FIT is an imperfect diagnostic test for colorectal disease and will miss some currently referred patients, who have CRC, other significant premalignant polyps, inflammatory bowel disease, and other non-colorectal cancer. FIT should be used in all patients at high risk of CRC to inform the need for, type, and timing of further investigation. Studies are needed to understand how best to optimise the benefits of FIT in the clinical context of patients fulfilling National Institute for Health and Care Excellence guideline 12 (NG12) referral criteria for suspected CRC.

## METHOD

### Study design

Ethical approval was obtained to conduct a prospective, blinded, multi-centre diagnostic accuracy study of FIT for clinical outcomes in patients referred with suspected CRC within the ‘2-week wait’ pathway from 25 April 2018 to 31 December 2019.

### Participants

Twelve secondary care providers across Yorkshire and Humber were involved in this study. It was conducted following the standards for the reporting of diagnostic accuracy studies guidelines.^17^ A referral proforma containing the NG12 referral criteria was used by GPs to access the ‘2-week wait’. At each site, a convenience series of patients attending dedicated ‘2-week wait’ colorectal outpatient or telephone clinics were consented for the study by a research nurse. The ‘convenience’ related to the availability of the research nurse rather than any patient characteristics. Patient symptoms and relevant medical history were recorded. While GPs were guided by the NG12 referral criteria, to ensure that the study was representative of the population currently being referred, a formal assessment of compliance was deliberately not undertaken.

### Test methods

The consenting process was independent of any decision by the responsible clinician to investigate the patient. Symptomatology, patient demographics, Index for Multiple Deprivation (IMD), use of non-steroidal anti-inflammatory drugs (NSAIDs), antiplatelet or anticoagulant therapy, relevant personal and family history, and baseline blood tests were recorded. The decision to investigate was made on clinical grounds at the discretion of the responsible clinician. Patients and clinicians were blinded to the FIT result throughout. Only patients undergoing full colonoscopy or CT colonography, or a lesser investigation (such as CT abdomen/pelvis with contrast or flexible sigmoidoscopy), were included in the data analysis.^18^ Relevant data and final diagnoses accessed from patient-management systems were stored anonymously on an electronic case report form. Significant premalignant polyps are defined as adenomatous or hyperplastic lesions with high-grade dysplasia, or one ≥10 mm in size or if there are a total of ≥5 subcentimetre polyps (excluding hyperplastic rectal polyps).^19^ OED includes IBD, microscopic colitis, radiation proctopathy, and those cases where the responsible clinician judged the referral diagnosis to be diverticular disease. IBD is reported separately from other OED in some statistical analysis. Asymptomatic, moderate diverticulosis, or that described as minor or mild was not included within OED. When no CRC, significant polyp, or OED diagnosis was made the diagnosis was reported as irritable bowel syndrome, haemorrhoidal bleeding, or iron deficiency (no cause found) as appropriate. For the purposes of the study this cohort has been grouped as ‘other functional diagnoses’.

### FIT analysis

Consenting patients collected a single faecal sample using an EXTEL HEMOAUTO MC collection device between their out-patient consultation and subsequent investigation. FIT analysis was performed using an automated turbidometric system, HM-JACKarc. Calibration was performed in line with the manufacturer’s instructions, and internal quality control samples provided by the manufacturer were analysed in each batch. The analytical coefficient of variation (% CV) between batches was 4.6% at a concentration of 27 µg Hb/g faeces and 3.6% at a concentration of 102 µg Hb/g faeces. External quality assessment samples from UK NEQAS were analysed regularly. The manufacturer’s quoted limit of quantitation of 7 µg Hb/g faeces (imprecision <10% CV), analytical range of 7–400 µg Hb/g faeces, and limit of detection of 2 µg Hb/g faeces were used in this study.

### Sample size

Using an expected CRC incidence of 3%–5%, this study aimed to recruit a minimum of 5000 patients in order to achieve a representative sample for this diagnostic accuracy study.^14^

### Statistical analysis

For the purposes of this study, the primary diagnostic accuracy analyses of FIT in detecting CRC and the secondary clinical outcomes were derived using receiver operating characteristics (ROC) curves. The point on the ROC curve that maximises both sensitivity and specificity was used to determine a statistically optimal cut-off value. Estimates of the area under the curve (AUC), sensitivity, specificity, and negative (NPV) and positive (PPV) predictive values were calculated (using the optimal cut-off value for the latter four measures) and presented alongside 95% confidence intervals (CIs). Since the optimisation of FIT may ultimately be determined by a composite of clinical factors beyond Youden’s index, the sensitivity, specificity, NPV, and PPV of FIT were also calculated for cut-offs of 2 (the limit of detection), 10, 30, 100, and 300 µg Hb/g faeces. The proportion of disease cases versus non-disease cases within different ranges of FIT were explored graphically.

## Results

### Participants

**Figure 1.**
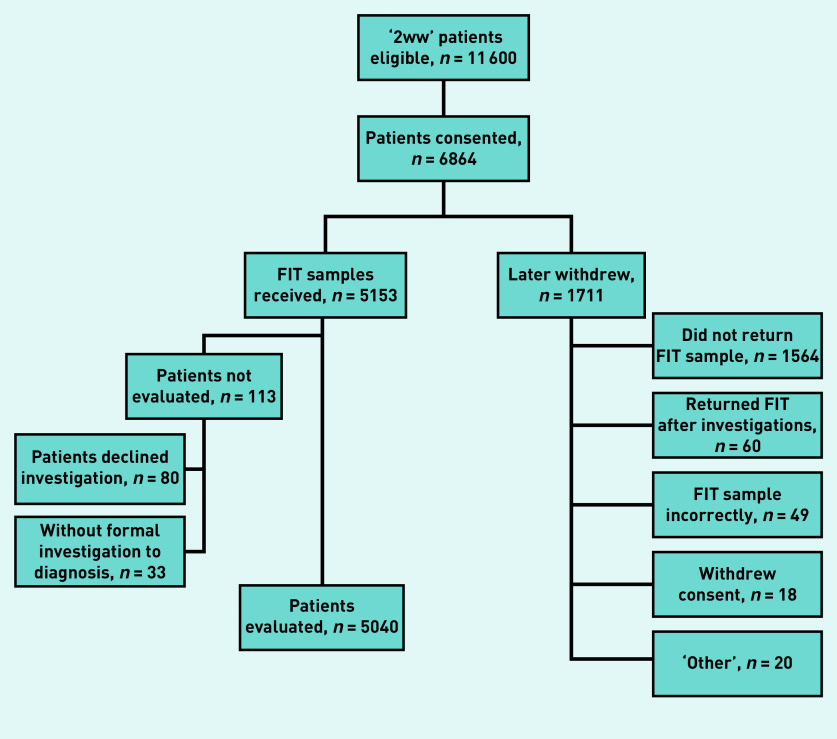
*Flow of participants from attendance in 2-week wait clinics for suspected CRC through to formal evaluation. 2WW = 2-week wait. CRC = colorectal cancer. FIT = faecal immunochemical test.*

In total, 5153 patients were recruited (Figure 1 and Supplementary Table S1). The mean age was 67.4 (standard deviation 11.7) years and 2852 (55.3%) of the patients were female. The most common presenting symptoms were diarrhoea (*n* = 1872; 36.3%), abdominal pain (*n* = 1746; 33.9%), and fresh rectal bleeding (*n* = 1721; 33.4%). Approximately 10% of patients had a family history of CRC, with 1389 (27.0%) using either antiplatelet therapy, anticoagulants, or NSAIDs (see Supplementary Tables S1 and S2). Of the 5153 recruited patients, 113 (2.2%) either declined or were not offered any formal investigations and were excluded from the primary and secondary analyses. Clinical and demographic details of those remaining 5040 patients with a primary or secondary outcome are presented in Supplementary Tables S3 and S4.

The most common investigations were colonoscopy (*n* = 3857; 76.5%), CT colonography (*n* = 751; 14.9%), and CT of the abdomen or pelvis (*n* = 1086; 21.5%) (see Supplementary Table S5). Final diagnoses were 3.0% CRC (*n* = 151), 4.1% significant premalignant polyps (*n* = 206), 2.0% IBD (*n* = 100), 15.3% OED (*n* = 771), 17.6% diminutive colorectal polyps (*n* = 682), 8.3% significant non-enteric disease (*n* = 418), and 53.8% other functional diagnoses (*n* = 2712).

### Primary analysis

#### Diagnostic accuracy of FIT for colorectal cancer

CRC was detected in 151 (3.0%) of the 5040 patients evaluated. An optimal cut-off value of 19 µg Hb/g faeces was determined giving a sensitivity of 85.4% (95% CI = 78.8% to 90.6%), a specificity of 85.2% (95% CI = 84.1% to 86.2%), a PPV of 15.1% (95% CI = 12.8% to 17.7%), and an NPV of 99.5% (95% CI = 99.2% to 99.7%) (Table 1 and Supplementary Table S6). The AUC was estimated to be 0.89 (95% CI = 0.86 to 0.92) (see Supplementary Figure S1). Using this threshold, 854 (16.9%) patients were considered to have a ‘positive FIT’, of whom 129 (85.4% of those with CRC) had CRC. By contrast, 4186 (83.1%) patients were considered to have a ‘negative FIT’ with 22 (14.6% of those with CRC) having CRC.

**Table 1.** Primary outcome analysis and subgroup analyses

	***N***	**Cases, *n* (%)**	**Optimal cut-off, µg/g**	**Sensitivity, % (95% CI)**	**Specificity, % (95% CI)**	**PPV, % (95% CI)**	**NPV, % (95% CI)**	**AUC (95% CI)**
**Primary outcome**								

All participants with formal investigations	5040	151 (3.0)	19	85.4 (78.8 to 90.6)	85.2 (84.1 to 86.2)	15.1 (12.8 to 17.7)	99.5 (99.2 to 99.7)	0.89 (0.86 to 0.92)

**Subgroup analyses**								

**Age, years**								

<60	1217	30 (2.5)	37	90.0 (73.5 to 97.9)	87.4 (85.4 to 89.3)	15.3 (10.4 to 21.5)	99.7 (99.2 to 99.9)	0.92 (0.88 to 0.96)
≥60	3823	121 (3.2)	19	83.5 (75.6 to 89.6)	85.4 (84.2 to 86.5)	15.7 (13.0 to 18.8)	99.4 (99.0 to 99.6)	0.88 (0.85 to 0.92)

**Sex**								
Male	2242	89 (4.0)	21	85.4 (76.3 to 92.0)	83.7 (82.0 to 85.2)	17.8 (14.3 to 21.7)	99.3 (98.8 to 99.6)	0.89 (0.86 to 0.93)
Female	2798	62 (2.2)	16	87.1 (76.1 to 94.3)	85.6 (84.2 to 86.9)	12.0 (9.2 to 15.4)	99.7 (99.3 to 99.9)	0.88 (0.82 to 0.93)

**Change in bowel habit**								
Yes	3467	89 (2.6)	16	85.4 (76.3 to 92.0)	85.8 (84.5 to 86.9)	13.6 (10.9 to 16.8)	99.6 (99.2 to 99.8)	0.89 (0.85 to 0.93)
No	1573	62 (3.9)	21	87.1 (76.1 to 94.3)	82.3 (80.2 to 84.2)	16.8 (12.9 to 21.3)	99.4 (98.7 to 99.7)	0.89 (0.84 to 0.93)

**Rectal bleeding**								
Yes	1912	77 (4.0)	37	90.9 (82.2 to 96.3)	83.2 (81.4 to 84.8)	18.5 (14.7 to 22.7)	99.5 (99.1 to 99.8)	0.90 (0.87 to 0.93)
No	3128	74 (2.4)	10	79.7 (68.8 to 88.2)	84.0 (82.6 to 85.3)	10.8 (8.3 to 13.7)	99.4 (99.0 to 99.7)	0.87 (0.82 to 0.92)

**Abdominal pain**								
Yes	1722	47 (2.7)	10	85.1 (71.7 to 93.8)	82.5 (80.6 to 84.3)	12.0 (8.7 to 16.0)	99.5 (99.0 to 99.8)	0.88 (0.83 to 0.93)
No	3318	104 (3.1)	37	85.6 (77.3 to 91.7)	88.4 (87.2 to 89.5)	19.2 (15.7 to 23.1)	99.5 (99.1 to 99.7)	0.90 (0.86 to 0.93)

**Weight loss**								
Yes	1093	38 (3.5)	13	89.5 (75.2 to 97.1)	83.1 (80.7 to 85.3)	16.0 (11.4 to 21.7)	99.5 (98.8 to 99.9)	0.88 (0.82 to 0.94)
No	3947	113 (2.9)	19	85.8 (78.0 to 91.7)	85.0 (83.8 to 86.1)	14.4 (11.9 to 17.3)	99.5 (99.2 to 99.7)	0.89 (0.86 to 0.93)

**ID anaemia^a^**								
Yes	559	34 (6.1)	21	82.4 (65.5 to 93.2)	81.5 (77.9 to 84.8)	22.4 (15.4 to 30.7)	98.6 (97.0 to 99.5)	0.87 (0.80 to 0.93)
No	3582	101 (2.8)	19	88.1 (80.2 to 93.7)	85.3 (84.0 to 86.4)	14.8 (12.0 to 17.9)	99.6 (99.3 to 99.8)	0.90 (0.87 to 0.93)

a *Number of patients and cases do not add up to 5040 and 151, respectively, owing to missing anaemia and ID status. AUC = area under curve. ID = iron deficiency. NPV = negative predictive value. PPV = positive predictive value.*

**Figure 2.**
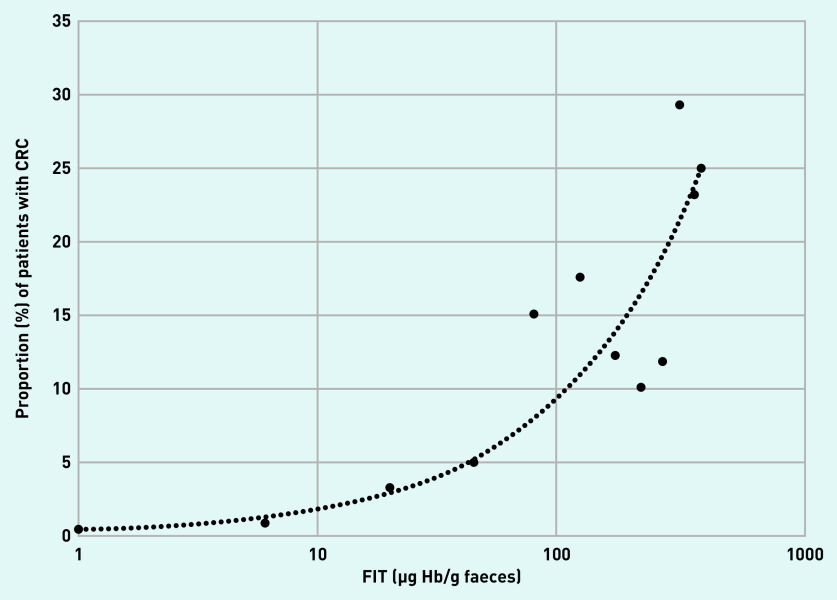
*Proportion of CRC at different FIT level (µg Hb/g faeces) ranges. Each data point represents the midpoint of consecutive FIT level ranges. Readings <2 are represented by a value of 1 and readings of ≥400 are represented by a value of 400. Line represents automatically fitted power trendline. CRC = colorectal cancer. FIT = faecal immunochemical test.*

The location of the CRC, whether right-sided, left-sided, or rectal did not alter the diagnostic accuracy of FIT. The sensitivity, specificity, PPV, and NPV of FIT for CRC at five different fixed positivity thresholds from 2–300 µg Hb/g faeces were determined, and the proportion of CRC based on different FIT ranges presented graphically (Table 2 and Figure 2). An exploratory analysis of the tumour stage of the TNM Classification of Malignant Tumours was available for 114 patients with CRC (see Supplementary Table S7). Of the 19 patients with CRC TNM staging and FIT ≤18 µg Hb/g faeces, 21.1% (*n* = 4) were T1, 31.6% (*n* = 6) T2, 26.3% (*n* = 5) T3, and 21.1% (*n* = 4) T4.

**Table 2.** Diagnostic accuracy of FIT in detecting the primary outcome of colorectal cancer at thresholds of 2, 10, 30, 100, and 300 µg Hb/g faeces

	**Sensitivity, % (95% CI)**	**Specificity, % (95% CI)**	**PPV, % (95% CI)**	**NPV, % (95% CI)**
**FIT ≥2 µg/g**	92.7 (87.3 to 96.3)	60.7 (59.3 to 62.1)	6.8 (5.7 to 8.0)	99.6 (99.3 to 99.8)
**FIT ≥10 µg/g**	87.4 (81.0 to 92.3)	80.9 (79.7 to 81.9)	12.4 (10.4 to 14.5)	99.5 (99.3 to 99.7)
**FIT ≥30 µg/g**	80.1 (72.9 to 86.2)	87.7 (86.8 to 88.6)	16.8 (14.1 to 19.7)	99.3 (99.0 to 99.5)
**FIT ≥100 µg/g**	66.2 (58.1 to 73.7)	92.7 (91.9 to 93.4)	21.8 (18.1 to 25.8)	98.9 (98.5 to 99.2)
**FIT ≥300 µg/g**	53.0 (44.7 to 61.1)	95.1 (94.5 to 95.7)	25.2 (20.5 to 30.3)	98.5 (98.1 to 98.8)

*FIT = faecal immunochemical test. NPV = negative predictive value. PPV = positive predictive value.*

#### Subgroup analyses: symptoms, demographics, and drugs

Subgroup analyses were performed across a range of demographics, symptoms, and the use of drugs to identify subgroup-specific FIT optimal cut-off values (Table 1 and Supplementary Table S6). There were no differences in the sensitivity of FIT in the subgroup analyses. However, the specificity of FIT differed in the following subgroups: change in bowel habit, constipation, abdominal pain, and drug use.

### Secondary analyses

#### Diagnostic accuracy of FIT for CRC, significant polyps, IBD, and all OED

**Figure 3.**
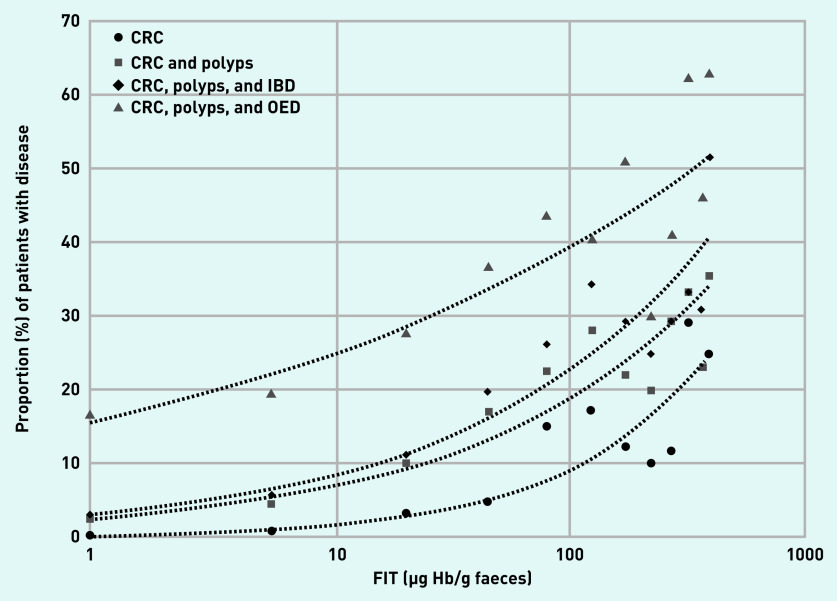
*Proportion of disease cases (CRC with significant premalignant polyps, IBD and all OED) at different FIT level (µg Hb/g faeces) ranges. Each data point represents the midpoint of consecutive FIT level ranges. Readings <2 are represented by a value of 1 and readings of ≥400 are represented by a value of 400. Lines represent automatically fitted power trendlines. CRC = colorectal cancer. FIT = faecal immunochemical test. IBD = inflammatory bowel disease. OED = organic enteric disease.*

In total, 342 (6.8%) patients had the secondary outcome of having either CRC or significant premalignant polyps, while 1147 (22.8%) had the secondary outcome of having either CRC, significant premalignant polyps, IBD, or OED (see Supplementary Table S7). The diagnostic accuracy of FIT in this setting was poorer, and is presented both at the optimal cut-off value for each secondary analysis group and at 19 µg Hb/g faeces (Table 3). Here 717 patients with secondary diagnoses (72.1%) were ‘FIT negative’. This represents 59.6% of the patients with significant premalignant polyps and 36.0% of the patients with IBD. The proportion of CRC or one of these secondary diagnoses based on the FIT range is presented graphically (Figure 3).

**Table 3.** Secondary outcome analyses

	***N***	**Cases, *n* (%)**	**Optimal cut-off, µg/g**	**Sensitivity, % (95% CI)**	**Specificity, % (95% CI)**	**PPV, % (95% CI)**	**NPV, % (95% CI)**	**AUC (95% CI)**
**Secondary outcomes**								
CRC or SPP	5040	342 (6.8)	7	69.6 (64.4 to 75.4)	78.9 (77.7 to 80.0)	19.3 (17.2 to 21.7)	97.3 (96.7 to 97.8)	0.79 (0.76 to 0.82)
CRC, SPP, or IBD^a^	5040	442 (8.8)	6	69.9 (65.4 to 74.2)	78.6 (77.4 to 79.8)	23.9 (21.6 to 26.4)	96.5 (95.8 to 97.0)	0.80 (0.77 to 0.82)
CRC, SPP, IBD, or OED	5040	1147 (22.8)	2	56.7 (53.7 to 59.6)	63.8 (62.2 to 65.3)	31.6 (29.5 to 33.6)	83.3 (81.9 to 84.6)	0.64 (0.62 to 0.66)
**Diagnostic accuracy of FIT in detecting each secondary outcome using a cut-off of 19 µg Hb/g faeces**								
CRC or SPP^a^				62.9 (57.5 to 68.0)	86.4 (85.4 to 87.4)	25.2 (22.3 to 28.2)	97.0 (96.4 to 97.5)	—
CRC, SPP, or IBD^a^				63.1 (58.4 to 67.6)	87.5 (86.5 to 88.4)	32.7 (29.5 to 35.9)	96.1 (95.5 to 96.7)	—
CRC, SPP, IBD, or OED^a^				35.7 (32.9 to 38.5)	88.6 (87.5 to 89.6)	47.9 (44.5 to 51.3)	82.4 (81.1 to 83.5)	—

a *Exploratory analysis. AUC = area under curve. CRC = colorectal cancer. FIT = faecal immunochemical test. IBD = inflammatory bowel disease. NPV = negative predictive value. OED = organic enteric disease. PPV = positive predictive value. SPP = significant premalignant polyps.*

#### Opportunistic and non-enteric diagnoses

Of the 206 patients with significant premalignant polyps, only 43.0% had symptoms of rectal bleeding or rectal mass; the remainder should be considered opportunistic findings. A further 682 patients were found to have opportunistic, low-risk premalignant polyps, 84.0% of whom had a FIT <19 µg Hb/g faeces. Significant non-enteric disease that required onward medical management was found in 418 (8.3%) additional patients, of whom 83 (19.9%) had non-colorectal cancers (see Supplementary Table S7).

## DISCUSSION

### Summary

This diagnostic accuracy study, recruiting over 5000 patients in a convenience series, represents as closely as pragmatically possible the population of adults seen within primary care with symptoms judged to be high-risk for CRC. The IMD seen across the 12 NHS Hospital Trusts in Yorkshire and Humber in this study broadly mirrors that in England overall, and includes a number of large conurbations with an ethnic diversity. Colonoscopy, CTC or flexible sigmoidoscopy and abdomino-pelvic CT were performed on 92.5% of patients.^19^ Only 33 patients were excluded from the evaluation as they underwent no secondary care investigation. This likely reflects the current clinical imperative of secondary care to investigate patients referred with suspected CRC. A statistically optimal cut-off value of 19 µg Hb/g faeces for CRC was determined using ROC curves, giving a sensitivity of 85.4% (95% CI = 78.8% to 90.6%) and specificity of 85.2% (95% CI = 84.1% to 86.2%). The negative predictive value at this cut-off value was 99.5% (95% CI = 99.2% to 99.7%) and the positive predictive value was 15.1% (95% CI = 12.8% to 17.7%).

### Comparison with existing literature

Previous smaller diagnostic accuracy studies quoted a sensitivity for CRC of very close to 100% for FIT.^20^^,^^21^ Subsequently, it became clear that dependent on the cut-off chosen, FIT will miss between 7% and 15% of patients with CRC.^22^^–^^24^ The sensitivity and specificity of FIT for CRC in this study aligns with the smaller studies that recruited patients fulfilling NG12 criteria.^16^^,^^25^ The authors’ previous study determined the optimal cut-off value for FIT to be ≥12 µg Hb/g faeces. In that study, however, faecal sampling into collection devices was performed in the laboratory instead of by the patient and this may have resulted in some pre-analytic haemoglobin degradation.^26^^,^^27^ The published study most comparable by design — since it too recruited patients exclusively referred through the ‘2-week wait’ for CRC and used an HM-JACKarc analyser — found a similar sensitivity and specificity of 84% and 93%, respectively.^25^ In Scotland, where the Scottish Intercollegiate Guidance Network guidance produces a different referral population from NICE, FIT has a similar diagnostic accuracy to this study.^21^^,^^28^^–^^30^ A similar sensitivity and specificity are obtained in the two other large diagnostic accuracy studies that have been conducted in England: the NICE FIT study and the qFIT pilot study. The recently published NICE FIT study reports an AUC for CRC of 0.93 (95% CI = 0.92 to 0.95) and an optimal cut-off value for FIT of 38 µg Hb/g faeces.^31^^,^^32^

### Strengths and limitations

This diagnostic accuracy study presents its findings in terms of the statistical optimisation of the sensitivity and specificity of FIT. The use of a statistically optimal cut-off value highlights the need for FIT to be considered as a tool by which to both minimise the risk of missing CRC, and to optimise the use of investigative resource within a constrained healthcare system. There is an inevitable trade-off between the two. Reconciling that trade-off is a major healthcare challenge. Ultimately, a detailed and comprehensive health economic analysis is required to determine the true clinical utility of FIT. This is beyond the scope of this study; however, recognising the complexity of this task, the data have also been presented with a range of cut-off values: 2 (the limit of detection), 10, 30, 100, and 300 µg Hb/g faeces.

Very little is yet known about the response of symptomatic patients and their clinicians to a FIT-based assessment, and the savings of investigative resource that might result. Therefore, it was deemed that using a statistical measure represented the appropriate starting point for that risk analysis. Using this approach, a high sensitivity and specificity for FIT is retained across age and sex, symptoms and signs, medicines use, and anaemia. The optimal cut-off value for people aged ≥60 years (19 µg Hb/g faeces) is lower than for those aged <60 years (37 µg Hb/g faeces), and is lower for females (16 µg Hb/g faeces) than for males (21 µg Hb/g faeces).^16^^,^^33^^–^^35^ Interestingly, and contrary to NICE diagnostics guidance 30 (DG30), it was found that FIT retained a high diagnostic accuracy in those with rectal bleeding, although the optimal cut-off value was higher in those with (37 µg Hb/g faeces) than without (10 µg Hb/g faeces) bleeding. It had previously been the authors’ experience with faecal calprotectin that the diagnostic accuracy of a faecal biomarker was preserved in the context of rectal bleeding.^36^ It was speculated that anal canal bleeding might coat rather than impregnate the faeces, and thereby not interfere with sampling from the centre of a formed faecal sample. Previous studies have also suggested that the diagnostic accuracy of FIT is lower in patients with anaemia, but this was not found to be the case in iron deficiency or iron deficiency anaemia.^21^^,^^23^ In line with the qFIT pilot study, the optimal cut-off value for those patients with abdominal pain was set lower at 10 µg Hb/g faeces.^32^ Lastly, only in the small subgroup of patients who had an abdominal mass did FIT achieve a sensitivity of 100% (see Supplementary Table S6). Otherwise, FIT inevitably misses CRC in a small number of patients. Alternative approaches to using FIT — such as applying the lowest possible cut-off — either the limit of quantitation (7 µg Hb/g faeces) or the limit of detection (2 µg Hb/g faeces) are unlikely to be effective in preventing missed CRC. They will improve the NPV of FIT by 0.1% at the cost of an inferior PPV, and so will more than double the burden imposed on investigative resource. This trade-off also applies at the cut-off value 10 µg Hb/g faeces, as currently recommended by NHS England in its specialty guides for patient management during the coronavirus pandemic.^37^ The imperfect nature of FIT at whatever cut-off value chosen reinforces the need for a formal, contextualised health economic analysis to determine a clinically — rather than necessarily statistically — optimal FIT cut-off value.^38^

### Implications for practice

Whatever acceptable balance of risk is ultimately arrived at, the authors believe that FIT must primarily be used to ‘democratise’ the CRC risk assessment.^39^ In future, a personalised, optimal FIT cutoff value can be generated as a ‘risk score’ for an individual patient, based on sex, age, symptoms and signs, drug history, and blood parameters. GP electronic requesting systems can be used to capture clinical indications for FIT requests, and this can be linked together with demographic data, other blood results, and FIT results in the laboratory information system. These data could be used in future by an automated algorithm to generate a personalised risk score to accompany or replace the numerical FIT result, and this risk score could then be reported along with recommendations on referral or management based on it.

That personalised CRC risk next needs to be incorporated into a personalised clinical assessment of the patient. This is the challenge. This study demonstrates that ‘FIT negative’ patients with NG12 criteria for suspected CRC have a CRC risk <0.5%. This may be lower than the prevalent risk of CRC in an equivalently aged asymptomatic population.^40^^,^^41^ But more than one in five of the patients referred had an OED that required prompt diagnosis and management, even if not within the ‘2-week wait’ timeframe. This included 14.2% of the ‘FIT negative’ patients, such as those with IBD, where early diagnosis has been shown to minimise complications and the need for surgery.^42^^,^^43^ In addition, another 8.3% of patients had significant non-enteric disease, including other cancers such as ovarian, pancreatic, and renal cancer. In total, non-CRC malignancies accounted for 35.5% of all the cancer diagnoses in this study.^44^ The opportunistic diagnosis of diminutive premalignant colorectal polyps represents a further cohort, currently undefined, of screening benefit for the population. Lastly, many of those with ongoing functional symptoms and haemorrhoidal bleeding will remain symptomatic within a population previously considered high-risk for CRC. In England, initial symptomatic treatment strategies are not currently offered in these patients, as they are in Scotland.^45^^–^^48^ While only 16.9% of ‘2-week wait’ patients referred in this study had a ‘positive FIT’, this will not represent the proportion of patients ultimately referred to secondary care in any future FIT-based pathway. The authors have previously estimated that 25% might be spared investigation by the use of a faecal biomarker and the resolution of symptoms.^49^

FIT can reduce CRC risk well below the 3% threshold on which the NG12 guidelines were devised. However, it is not currently known what alternative management strategies are required to support optimal patient and clinician decisionn making on the need for, type, and timing of investigations.^50^^–^^53^ Investigation is not risk free, and for many frail patients that risk will now exceed any benefit that could be derived from early diagnosis of disease.^54^^,^^55^ If FIT could spare unnecessary investigation and redirect resource to other diagnostic pathways such as the BCSP, there could be a net significant health economic benefit for the wider population.^9^^–^^11^ Those NG12 patients with CRC missed because of a ‘negative FIT’ would be offset by the increased number of detected participants with CRC in the BCSP. FIT has a high diagnostic accuracy for CRC, and should be used in the clinical assessment of all patients fulfilling the NICE NG12 criteria for suspected CRC.^37^^,^^55^^,^^56^ Patients in whom FIT sufficiently alters that risk assessment should no longer be investigated within the ‘2-week wait’. However, it is important to recognise that FIT will miss some patients with CRC, OED, and non-gastrointestinal pathology currently identified using NG12 criteria. FIT allows for a redesign of the current, largely symptom-based, referral process defined in NG12 into a novel, risk-based decision-making pathway for the care for patients with abdominal symptoms. If properly developed and applied, the net benefit of using FIT as an alternative to symptom-based referral criteria could be the optimised early diagnosis of CRC, and reduced morbidity and mortality for the whole population.
